# Repression of the Type VI Secretion System (T6SS) During Biofilm Formation Provides a Fitness Advantage for *Vibrio cholerae*

**DOI:** 10.3390/microorganisms14092024

**Published:** 2026-09-11

**Authors:** Stephan P. Ebenberger, Joao P. Pombo, Alexander Rechberger, Dominik Fleischhacker, Stefan Schild

**Affiliations:** 1 Institute of Molecular Biosciences, University of Graz, Humboldtstrasse 50, 8010 Graz, Austria; stephan.liegl@uni-graz.at (S.P.E.); joao.pombo@umu.se (J.P.P.); a.rechberger@lmu.de (A.R.); dominik.fleischhacker@uni-graz.at (D.F.); 2 BioTechMed Graz, Austria; 3 Field of Excellence Biohealth, University of Graz, 8010 Graz, Austria; 4 Austrian Agency for Health and Food Safety (AGES), Institute for Medical Microbiology and Hygiene, Beethovenstraße 6, 8010 Graz, Austria

**Keywords:** biofilm, type VI secretion system, *Vibrio cholerae*, gene repression, predator, prey

## Abstract

The type VI secretion system (T6SS) is a contact-dependent bacterial weapon to inject hazardous proteins into competitors and can be pivotal for bacterial fitness. Throughout the lifecycle of *Vibrio cholerae*, beneficial and detrimental activities of the T6SS have been described, but its role in biofilm-associated interactions remains poorly understood. Here, we show that biofilm formation is accompanied by significant repression of T6SS genes. Expression analyses across multiple *V. cholerae* isolates revealed consistent downregulation of the major T6SS gene cluster in biofilm-derived cells relative to planktonic cultures. Consistent with this biofilm-dependent repression, a loss of the T6SS did not affect *V. cholerae* biofilm formation. Notably, biofilm-derived *V. cholerae* wild-type isolates and their isogenic T6SS-deletion mutants displayed comparable resistance to attacks by T6SS-reactive *Pseudomonas aeruginosa*. These findings suggest that T6SS repression during biofilm growth provides a fitness advantage for *V. cholerae* by limiting susceptibility to T6SS-mediated counterattacks from neighboring heterologous T6SS^+^ predatory species. Our results, therefore, identify conditional T6SS silencing as a potential adaptive strategy that promotes survival within multispecies microbial communities, particularly in environments containing bacteria with highly active or more potent T6SS machineries.

## 1. Introduction

*Vibrio cholerae* is a facultative human pathogen that alternates between aquatic reservoirs and the human gastrointestinal tract, where it causes the severe secretory diarrheal disease cholera. Outside the host, *V. cholerae* persists in aquatic environments by forming surface-associated biofilms that promote environmental survival and transmission [1,2]. Throughout both environmental and host-associated lifestyles, bacteria encounter numerous biotic stressors, including predation and competition from conspecific and heterologous microorganisms. To cope with these challenges, bacteria have evolved a wide range of offensive, defensive, and evasive strategies. Among these, the type VI secretion system (T6SS) represents a widespread contact-dependent mechanism employed by Gram-negative bacteria to interact with neighboring cells. Approximately one quarter of all Gram-negative bacterial species encode genes associated with a T6SS, underscoring its importance in bacterial ecology and adaptation [3]. The T6SS functions as a contractile nanomachine that delivers toxic effector proteins into neighboring prokaryotic and eukaryotic cells, mediating interbacterial competition, defense against predators, and, in some species, contributing to virulence during host colonization [4].

The T6SS of *V. cholerae* is a multicomponent contractile apparatus that exhibits structural and functional homology to the tail machinery of contractile bacteriophages [5,6]. Assembly of the secretion apparatus is initiated by the formation of a membrane-associated core complex composed of VasD, VasF, and VasK, which anchors the system across the bacterial cell envelope [7]. Several cytoplasmic proteins, including HisF, VasA, VasB, VasE, and VasJ, are associated with this membrane complex through specific protein-protein interactions and contribute to the assembly process [8,9,10]. From this platform, an inner tube consisting of stacked hexameric Hcp rings polymerizes and is enveloped by a contractile sheath composed of VipA and VipB [5,11,12]. The distal end of the Hcp tube is capped by a VgrG spike, frequently sharpened by PAAR-containing proteins, which together form the puncturing device required for effector translocation into target cells [13,14,15]. Upon activation, rapid contraction of the sheath propels the Hcp tube and associated effectors out of the attacker cell and into the target cells [16]. Subsequently, the ATPase ClpV disassembles and recycles the contracted VipA/VipB sheath, thereby enabling multiple rounds of T6SS assembly and firing [11,17]. This dynamic cycle underlies the diverse functions of the T6SS in microbial interactions.

In *V. cholerae*, the T6SS genes are genetically organized into one main cluster (VC_A0105-VC_A0124) and up to five additional genomic regions known as auxiliary clusters [18]. Auxiliary cluster 1 (VC_A0017-VC_A0021) and auxiliary cluster 2 (VC_1415-VC_1419) are found in all *V. cholerae* genomes analyzed to date, while auxiliary clusters 3 to 5 show a more variable distribution and are present in some isolates [16,18,19,20]. The main cluster is located on chromosome II and encodes the majority of the structural components. The auxiliary clusters 1 and 2 are located on chromosomes I and II, respectively. Each of them encodes an almost identical *hcp* gene, either of which is sufficient to form the inner tube, *vgrG* genes, unique effector sets, and their cognate immunity proteins [19,21].

In *V. cholerae*, the T6SS confers fitness advantages across multiple ecological niches. In nutrient-limiting environments, where intense competition for resources occurs, the T6SS mediates antibacterial effectors into neighboring cells, eliminating competitors and promoting access to ecological niches and nutrients [22]. T6SS-dependent killing can additionally facilitate horizontal gene transfer through the release and uptake of genetic material from lysed cells, contributing to the acquisition of novel effector-immunity modules and shaping the spatial organization of microbial communities [23,24,25,26,27,28]. Beyond interbacterial competition, the T6SS provides protection against natural predators of *V. cholerae*, including amoebae and other protozoa, by delivering anti-eukaryotic effectors that impair predator viability and grazing activity [16,29]. During host colonization, T6SS activity enhances intestinal fitness in animal models by reducing competition from resident microbiota [26,30,31]. Furthermore, several T6SS effectors, including VgrG-1, directly target eukaryotic cells by disrupting cytoskeletal architecture, modulating immune responses, inducing inflammation, and altering gut peristalsis, thereby promoting displacement of commensal bacteria and facilitating colonization by *V. cholerae* [29,32,33,34,35].

Despite its ecological and pathogenic advantages, T6SS activity can also impose substantial fitness costs. Engagement in T6SS-mediated interactions may trigger retaliatory responses from neighboring bacteria possessing a more potent T6SS, a phenomenon referred to as “tit-for-tat” behavior [36,37]. Such counterattacks result in the death of the initiating cell population, highlighting the inherent tradeoff between offensive capabilities and vulnerability during interbacterial competition. Consequently, tight conditional regulation of T6SS activity seems essential to promote optimal fitness during diverse stages of the *V. cholerae* lifecycle. Consistent with this observation, our previous work identified two genes within the main T6SS cluster, VC_A0106 and VC_A0115, to be repressed during biofilm formation [38]. This finding suggested that T6SS expression levels may be more dynamic and context-dependent than previously recognized and raised the possibility that modulation of T6SS activity contributes to adaptation across different growth states.

In the present study, we comprehensively investigated the expression regulation of multiple T6SS-associated genes representing both the main and auxiliary T6SS clusters under planktonic and biofilm conditions across several *V. cholerae* isolates. Furthermore, we assessed the functional consequences of T6SS regulation during interactions with the bacterial competitors *Pseudomonas aeruginosa* and *Enterobacter cloacae*. *P. aeruginosa* delivers a potent T6SS-mediated counterattack to neighboring heterologous T6SS^+^ bacterial aggressors, resulting in T6SS-dependent killing of *V. cholerae* [36,37]. In contrast, *E. cloacae* served as a negative control, as T6SS expression is downregulated during LB cultivation, resulting in no detectable T6SS-dependent retaliatory activity [39]. Our results confirm that genes within the main T6SS cluster are silenced during biofilm formation in several *V. cholerae* isolates. Loss of the T6SS does not affect biofilm formation capacity. Remarkably, however, reduced T6SS expression enhances resistance of *V. cholerae* to T6SS-dependent attacks by competing bacteria. These findings reveal a previously unrecognized evasive strategy that promotes coexistence with potential competitors within surface-associated communities. Collectively, this study highlights the complex and conditional regulation of the T6SS in *V. cholerae* and demonstrates that modulation of T6SS activity provides adaptive advantages across distinct environmental conditions. Repression of the T6SS during biofilm growth may simultaneously reduce the energetic costs associated with secretion system deployment and minimize susceptibility to retaliatory attacks from neighboring microorganisms, thereby promoting survival within competitive polymicrobial communities.

## 2. Materials and Methods

### 2.1. Bacterial Strains and Culture Conditions

The strains and plasmids used in this study are listed in Table 1, and oligonucleotides (Microsynth AG, Balgach, Switzerland; Greiner Bio-One GmbH, Frickenhausen, Germany) are listed in Table 2. *Escherichia coli* strains DH5α*λpir* were used for cloning and *E. coli* SM10*λpir* for conjugation into *V. cholerae* strains, respectively. Unless otherwise noted, strains were grown in Lysogeny Broth (LB, pH = 7) at 37 °C with aeration (180 rpm) or on LB agar plates at 37 °C. Biofilm formation was achieved with LB (pH = 7) at 24 °C. Antibiotics (New England Biolabs, Ipswich, MA, USA) and other chemicals were used in the following final concentrations: streptomycin (Sm), 100 μg mL^−1^; ampicillin (Ap), 100 μg mL^−1^ or 50 μg mL^−1^ in combination with other antibiotics; kanamycin (Km), 50 μg mL^−1^; sucrose, 10% (*w*/*v*).

### 2.2. Generation of Genetically Altered Strains and Reporter Strains

Unless stated otherwise, the extraction of genomic DNA, PCRs, and purification of plasmids or PCR products were carried out as previously described [38]. QIAprep Spin Miniprep Kit and the QIAGEN Plasmid Midi Kit were used for plasmid isolations, while the QIAquick Gel extraction and QIAquick PCR Purification kits were used for purifying DNA fragments. PCR reactions for subcloning or generation of splicing by overlap extension (SOE)-PCR fragments were done using the Q5^®^ High-Fidelity DNA Polymerase (New England Biolabs, Ipswich, MA, USA), while the Taq DNA Polymerase (New England Biolabs, Ipswich, MA, USA) was used for all other PCRs.

Construction of in-frame T6SS deletion mutants in *V. cholerae* was performed by suicide vector mutagenesis using pΔT6SS, a derivative of pCVD442, as described by Donnenberg and Kaper [49]. To generate pΔT6SS, ~800 bp PCR fragments located upstream of VC_A0105 and downstream of VC_A0124 [corresponding to the whole main cluster of T6SS in *V. cholerae* [1]] were amplified using the oligonucleotide pairs T6SS_SacI_1 and T6SS_2 as well as T6SS_3 and T6SS_SalI_4, using chromosomal DNA from *V. cholerae* E7946 as template (Table 1). Both fragments were connected by a splicing overlap oligonucleotide, and the resulting SOE-PCR fragment was digested with the appropriate restriction enzyme (New England Biolabs, Ipswich, MA, USA) indicated by the name of the oligonucleotide and then ligated into SacI/SalI-digested pCVD442 [51]. Ligation products were transformed into electrocompetent *E. coli* DH5α*λpir* and Ap-resistant (ApR) colonies were analyzed for the correct constructs by PCR. To obtain deletion strains, *E. coli* SM10*λpir* was transformed with pΔT6SS, and the plasmid was conjugated into the respective *V. cholerae* strains. Exconjugants were selected on LB-Sm/Ap agar plates. Sucrose selection was used to obtain ApS colonies, and chromosomal deletions were confirmed by multiplex PCR. Since the T6SS main cluster spans ~ 25 kb, amplification of the original chromosomal region with a single primer pair is not feasible. Therefore, two primer pairs were used: T6SS_SacI_1/T6SS_SalI_4, which yields a ~ 1.6 kb fragment in the deletion mutant, and T6SS_col_1/T6SS_col_2, which amplifies a 473 bp internal fragment present only in the original chromosome. This strategy allows clear discrimination between deletion mutants and their originating strain (Table 2).

Derivatives of pGP*phoA* were constructed to obtain chromosomal *phoA* fusions to respective genes, as *phoA* acts as a useful genetic marker in *V. cholerae*. Promoterless *phoA* was used for the construction of VC_A0106::*phoA*, VC_A0124::*phoA*, VC_A0017::*phoA*, and VC_1418::*phoA* fusions. The respective gene fragments were amplified by PCR using oligonucleotide pairs VC_A0106_SacI and VC_A0106_KpnI, VC_A0124_SacI and VC_A0124_KpnI, VC_A0017_SacI and VC_A0017_KpnI, or VC_1418_SacI and VC_1418_KpnI (Table 2). The PCR products were digested with the restriction enzyme indicated by the oligonucleotide name, ligated with similarly digested pGP*phoA*, and transformed into *E. coli* DH5α*λpir* resulting in plasmids pGP*phoA*-VC_A0106, pGP*phoA*-VC_A0124, pGP*phoA*-VC_A0017 and pGP*phoA*-VC_1418 (Table 1). Correct constructs were verified with colony PCR. Validated plasmids were first transformed into *E. coli* SM10*λpir* and mobilized into the appropriate *V. cholerae* via conjugation. Exconjugants harboring the chromosomal *phoA* fusion were selected on LB-Sm/Ap agar plates as previously described [50].

### 2.3. Biofilm Assays

Biofilms in microtiter plates were assayed by crystal violet staining as previously described [52]. Briefly, the respective strains were grown overnight in LB, adjusted to an OD_600_ of 0.001 using fresh LB (pH = 7), and transferred to a 96-well microtiter plate (U bottom, Sterilin) with 130 µL per well and incubated for 48 h at 24 °C. Wells were then rinsed using a microplate washer (Fluido2, Anthos Mikrosysteme GmbH, Friesoythe, Germany); biofilm was stained with 0.1% (*w*/*v*) crystal violet, solubilized in 96% (*v*/*v*) ethanol, and the OD_595_ was measured (microplate reader: SPECTROstar Nano, BMG Labtech, Ortenberg, Germany) to quantify the amount of biofilm in each well.

### 2.4. Alkaline Phosphatase Assay

To determine the enzymatic activities for the *phoA*-fusions, alkaline phosphatase assays were performed as described previously [53]. In the case of planktonic cultures, the respective bacterial strains were grown overnight at 37 °C with aeration (180 rpm) before the bacterial cultures were harvested and subjected to the alkaline phosphatase assays [46,52]. For the preparation of biofilm-derived bacterial cultures, the respective bacterial strains were grown overnight in LB using a modified protocol based on the method previously described [52,54]. In brief, the overnight cultures were diluted to an initial OD_600_ of 0.001 in 3 mL of fresh LB medium (pH = 7.0) in sterile 15 mL conical tubes. The cultures were incubated statically for 48 h at 24 °C to allow biofilm formation on the inner surface of the tubes. Following incubation, planktonic cells were carefully removed, and the biofilms were gently washed once with fresh medium to remove residual non-adherent cells. The biofilm-associated cells were subsequently harvested by vigorous vortexing and repeated pipetting to disrupt the biofilm and release the attached cells. The resulting biofilm-derived cell suspensions were then subjected to the alkaline phosphatase assay. The activities were expressed in Miller units: (OD_405_ × 1000)/(OD_600_ × 0.96 × ∆t).

### 2.5. Killing Assay

Bacterial killing assays were performed as previously described with slight modifications [55]. For planktonic-derived *V. cholerae* cultures, bacterial strains were grown overnight at 37 °C. *V. cholerae* (prey) and *P. aeruginosa* (predator) or *E. cloacae* (competitor control) were harvested and mixed in a 1:10 ratio (prey: predator or prey: competitor control). 25 µL of the mixed bacterial culture was spotted onto ambient temperature-adjusted LB agar plates and incubated at 37 °C for 2 h. Afterward, bacteria were harvested and resuspended in 1 mL LB-Sm. Appropriate dilutions were plated on LB/Sm agar plates to determine the CFU of *V. cholerae* acting as prey.

In the case of biofilm-derived *V. cholerae* cultures, the respective *V. cholerae* were grown overnight in LB and adjusted to an OD_600_ of 0.001 using fresh LB. Bacterial suspensions were transferred to 50 mL conical tubes and incubated at 24 °C for 24 h to allow biofilm formation. After 24 h at 24 °C, cells attached in biofilms were gently washed twice with LB (pH = 7) to remove planktonic cells, before *P. aeruginosa* or *E. cloacae* were gently added to the *V. cholerae* biofilm. To do so, *P. aeruginosa* or *E. cloacae* were grown overnight in LB (pH = 7) and adjusted to an OD_600_ of 0.02 using fresh LB (pH = 7). The mixtures were incubated at 24 °C for an additional 24 h, before biofilms were gently washed twice with LB (pH = 7) to remove planktonic cells. The remaining biofilm was dissolved by vortexing and water-bath sonication as previously reported [56]. Appropriate dilutions were plated on LB/Sm agar plates to determine the CFU of *V. cholerae*.

For experiments with dispersed biofilms, the biofilm was allowed to grow as described above and subsequently dispersed by vortexing and treatment in an ultrasonic water bath as described previously [52,57]. This protocol was previously established and results in biofilm dispersal yielding predominantly small cell aggregates of approximately 50–100 cells without significant cell lysis or cell variability, which was assessed by several means including CFU measurements, plating, and direct counts by microscopy and general microscopical analyses as previously reported [52,57]. *P. aeruginosa* was added to the dispersed biofilm of *V. cholerae* as described above for intact biofilms. The mixtures were then incubated at 24 °C for an additional 24 h before determining the CFU of *V. cholerae* by plating appropriate dilutions on LB/Sm agar.

### 2.6. Statistical Analysis

Unless stated otherwise, the data are presented as median with interquartile range (IQR). Data were analyzed using the Mann–Whitney U test for single comparisons or the Kruskal–Wallis test, followed by post hoc Dunn’s correction, for multiple comparisons. GraphPad Prism version 10 was used for all statistical analyses. Differences were considered significant for *p* < 0.05.

## 3. Results

### 3.1. Reduced Expression of T6SS Genes During Biofilm Growth in V. cholerae

To determine whether expression of the main T6SS is regulated during biofilm growth, we quantified the expression levels of selected T6SS-associated genes under planktonic and biofilm conditions. Previous studies have demonstrated that T6SS expression and regulation can differ among *V. cholerae* strains [1,58]. To assess whether biofilm-dependent regulation is broadly conserved across genetically diverse *V. cholerae* isolates, we included the toxigenic O1 El Tor strain E7946, the non-toxigenic non-O1/non-O139 strain V52, the toxigenic O139 strain AI4450, and the non-toxigenic O1 El Tor strain VC2740-80 in our analyses.

**Figure 1:**
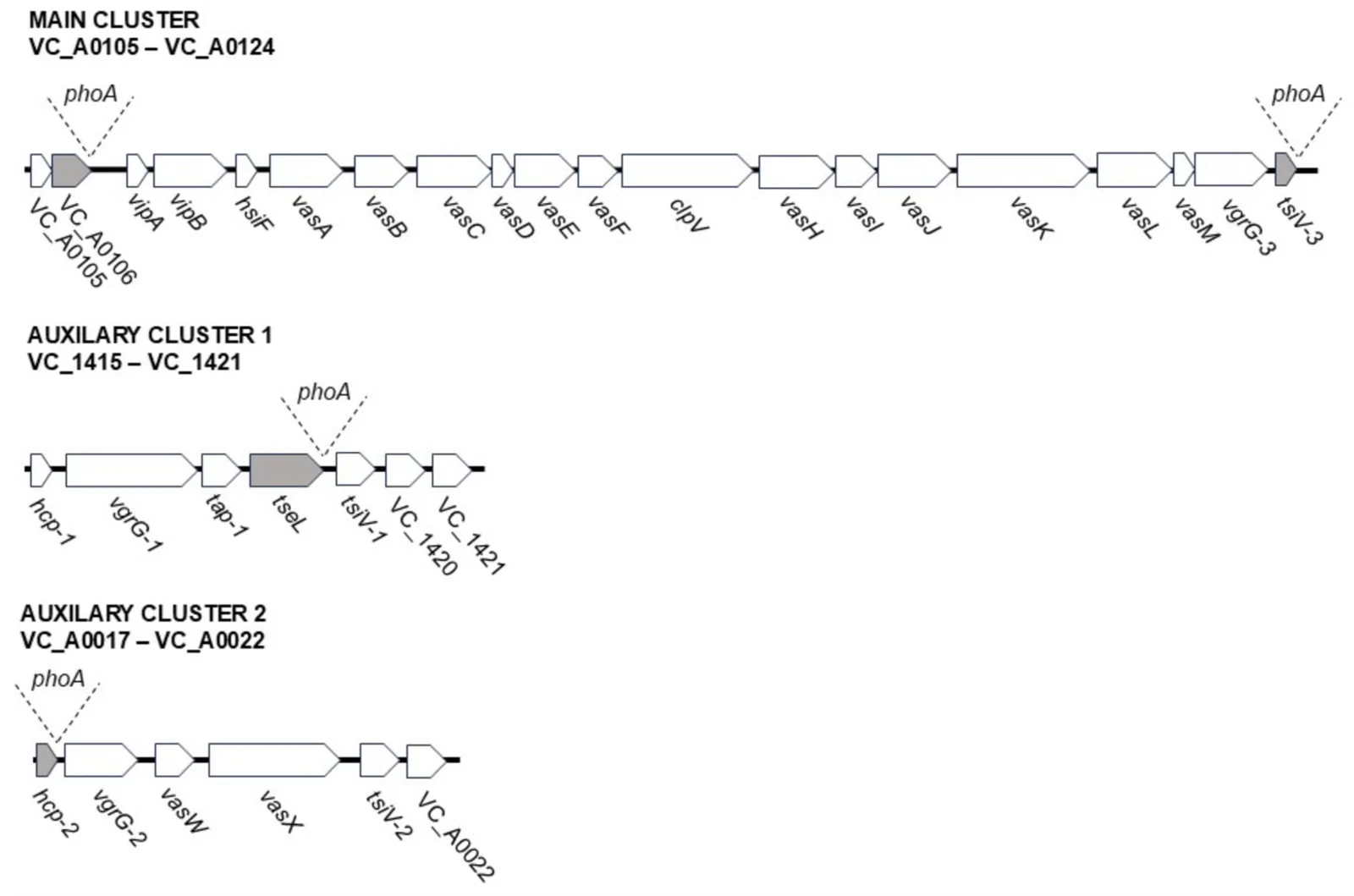
Genetic organization of the main and two auxiliary T6SS clusters in *V. cholerae*. Insertion sites of a promoterless *phoA* cassette in the main, auxiliary cluster 1, and auxiliary cluster 2 encoding the T6SS machinery are indicated by dotted lines. The upstream T6SS genes of each insertion are highlighted in gray, indicating the individual fusion constructs of this study. Genetic organization is adapted from Joshi et al. [1].

To investigate T6SS regulation, chromosomal reporter fusions of a promoterless *phoA* cassette to genes of the main, auxiliary cluster 1, and auxiliary cluster 2 were constructed to assess their expression level. Specifically, two genes from the main T6SS cluster, i.e., VC_A0106 and VC_A0124 (*tsiV-3*), one gene from the auxiliary cluster 1, i.e., VC_1418 (*tseL*), and one gene from the auxiliary cluster 2, VC_A0017 (*hcp-2*), were selected [18] (Figure 1). This approach enabled comprehensive coverage of all T6SS clusters identified in sequenced *V. cholerae* isolates to date, captured several previously described upstream and internal promoters, and included clusters distributed across both chromosomes [16,19,20,59]. The corresponding constructs (VC_A0106::*phoA*, VC_A0124::*phoA*, VC_1418::*phoA*, and VC_A0017::*phoA*) resulted in chromosomal fusions in the diverse *V. cholerae* isolates. Thus, PhoA activities were used as a proxy for the expression of the individual T6SS operons in the respective reporter strains, allowing comparison between the different *V. cholerae* isolates and identification of differential regulation between planktonic and biofilm conditions.

**Figure 2:**
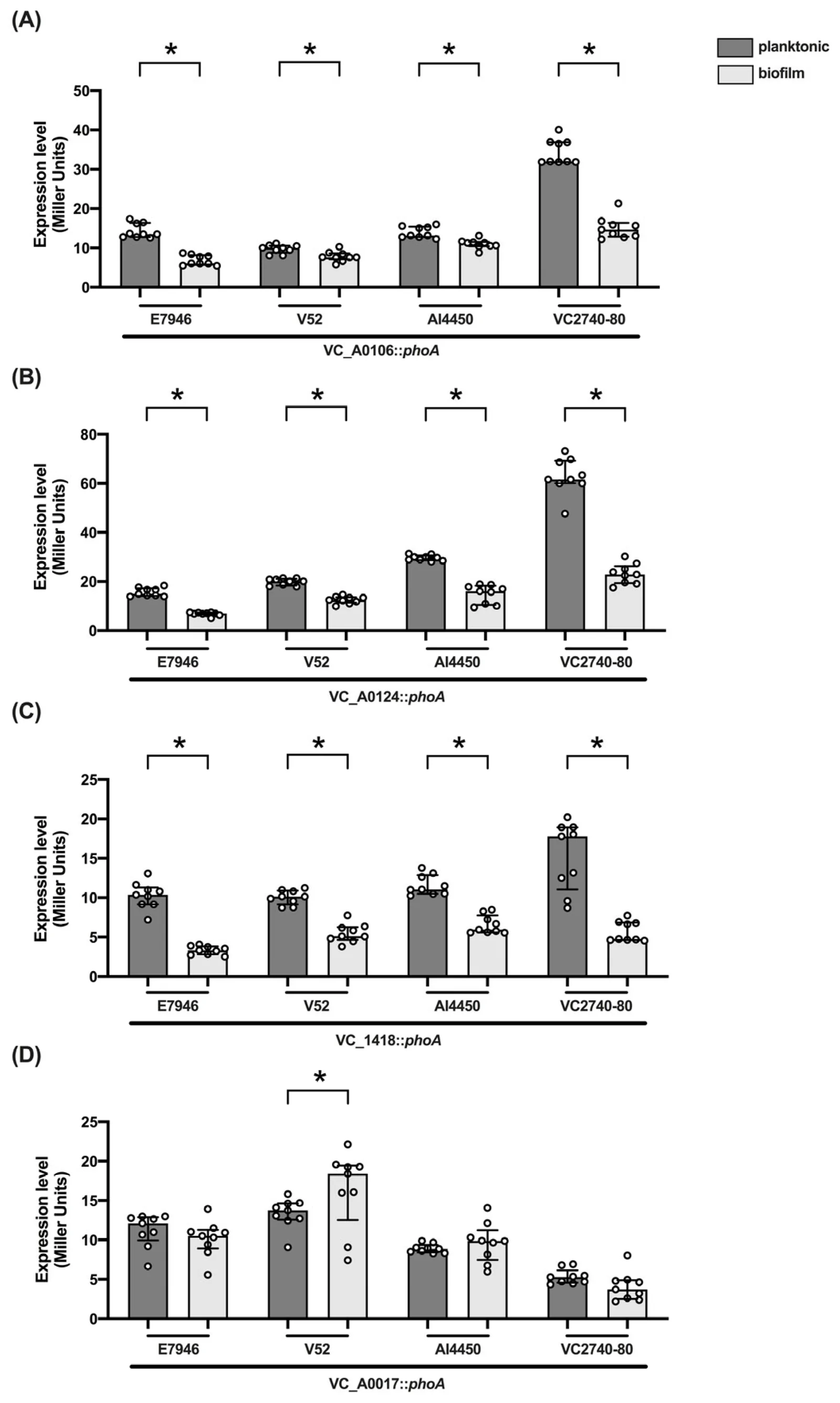
Expression levels of genes from the main and auxiliary T6SS clusters. Reporter strains of *V. cholerae* E7946, V52, AI4450, and VC2740-80 were constructed using the pGP*phoA*-derivative, resulting in chromosomal fusions of the promoter regions of VC_A0106, VC_A0124, VC_1418, and VC_A0017 to a promoterless *phoA* cassette. PhoA activities indicating the individual expression levels of (**A**) VC_A0106, (**B**) VC_A0124, (**C**) VC_1418, and (**D**) VC_A0017 were measured in “Miller units” from planktonic cultures (dark grey) and from biofilm cultures (light grey). Shown are the medians ± IQR of nine independent measurements. An asterisk indicates differences relative to the planktonic cells compared to the biofilm cells (*, *p* < 0.05 using a Mann–Whitney U test).

The expression of VC_A0106, previously reported to be downregulated during biofilm growth, was significantly reduced under biofilm conditions compared with planktonic-derived cultures for all four *V. cholerae* isolates [38]. Among the isolates tested, VC2740-80 exhibited the highest VC_A0106 expression levels during planktonic growth (Figure 2A). A similar pattern was observed for the second main cluster *phoA*-fusion to VC_A0124, which showed significantly lower expression in biofilm-derived cells compared to their planktonic cultures for all four *V. cholerae* isolates (Figure 2B).

To determine whether this regulatory pattern extended to T6SS auxiliary clusters 1 and 2, expression levels of VC_1418 (*tseL*) and VC_A0017 (*hcp-2*) were analyzed. Expression of VC_1418 mirrored that of the main T6SS cluster genes, with significantly higher expression detected under planktonic conditions in all four *V. cholerae* isolates tested (Figure 2C). In contrast, the expression level of VC_A0017 showed isolate-dependent regulation; no significant differences between planktonic and biofilm conditions were observed for E7946, AI4450, and VC2740-80. However, VC_A0017 expression levels in isolate V52 were slightly but significantly increased during biofilm growth compared to planktonic conditions (Figure 2D).

To exclude that the observed reduction in T6SS expression reflects a general decrease in gene expression during biofilm growth, expression of the non-T6SS gene *ompU* was analyzed across the same *V. cholerae* isolates. The expression of *ompU* exhibited an isolate-specific pattern, with increased, decreased, or unchanged expression under biofilm conditions (Figure S1). Thus, in contrast to the consistent reduction observed for the main T6SS cluster and auxiliary cluster 1, no uniform decrease in *ompU* expression was observed during biofilm growth. This is consistent with previous reports showing that gene expression during biofilm development is regulated in a gene-specific manner rather than being uniformly downregulated [52].

Collectively, these findings indicate that genes located within the main T6SS cluster and auxiliary cluster 1 are repressed during biofilm formation across a diverse set of *V. cholerae* isolates. In contrast, genes associated with auxiliary T6SS cluster 2 exhibit either unchanged expression levels or a modest increase in expression during biofilm formation. This pattern suggests that the regulation of auxiliary cluster 2 is less conserved among *V. cholerae* isolates and may be governed by distinct regulatory mechanisms that operate independently of those controlling the main T6SS cluster and auxiliary cluster 1. Importantly, the significant reduction in expression of the majority of T6SS genes, including those encoding core structural components of the protein secretion machinery, indicates that *V. cholerae* suppresses T6SS activity during biofilm formation.

### 3.2. Loss of the T6SS Does Not Affect Biofilm Formation in V. cholerae

**Figure 3:**
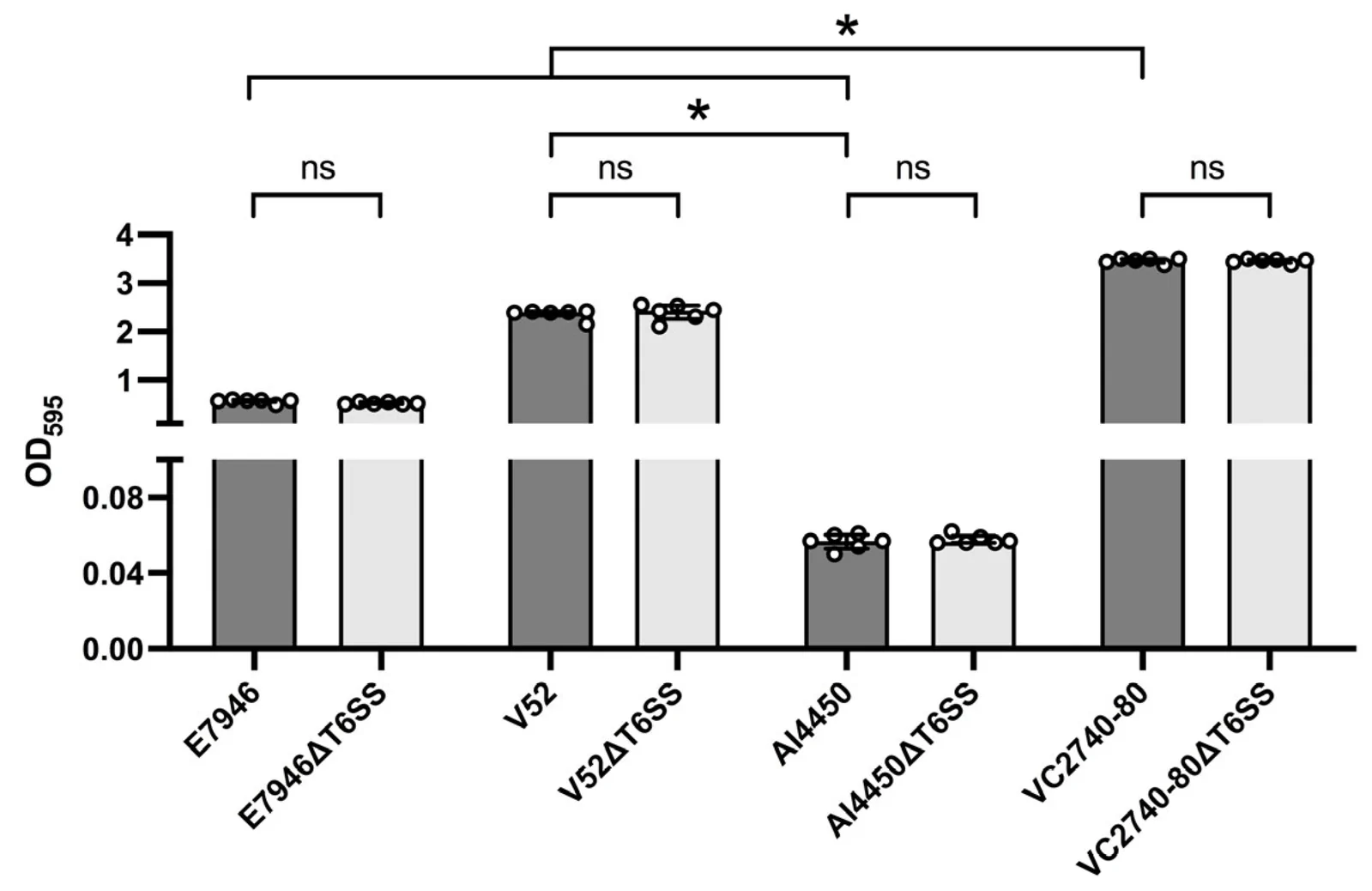
Deletion of the T6SS does not impact biofilm formation in *V. cholerae*. Biofilm formation capacities under biofilm conditions of different *V. cholerae* T6SS^+^ wild-type isolates (dark grey), and their isogenic ∆T6SS deletion mutants (light grey) were quantified by crystal violet staining and subsequent determination of the OD_595_. Shown are the medians ± IQR of six independent measurements. Statistical differences between the different wild-type isolates were analyzed via a Kruskal–Wallis test with corrected Dunn’s multiple comparison (*, *p* ≥ 0.05). No significant differences could be found between the wild-type and isogenic ∆T6SS deletion mutants (ns, *p* < 0.05 using a Mann–Whitney U test).

To assess whether T6SS activity contributes to biofilm development, biofilm formation was compared between T6SS^+^ *V. cholerae* isolates and their respective isogenic ∆T6SS deletion mutants. No significant differences in biofilm formation capacity were detected between mutants and their wild-type isolates, indicating that the absence of the T6SS does not impair biofilm development (Figure 3).

Despite the absence of T6SS-dependent effects, substantial strain-specific differences in biofilm-forming ability. The *V. cholerae* isolates E7946 and AI4450 showed comparatively low biofilm production, whereas V52 formed a larger amount of biofilm. Among the four isolates tested, VC2740-80 displayed the highest biofilm-forming capacity (Figure 3).

### 3.3. T6SS Activity Increases Susceptibility of V. cholerae to P. aeruginosa-Mediated Killing Under Planktonic Conditions

**Figure 4:**
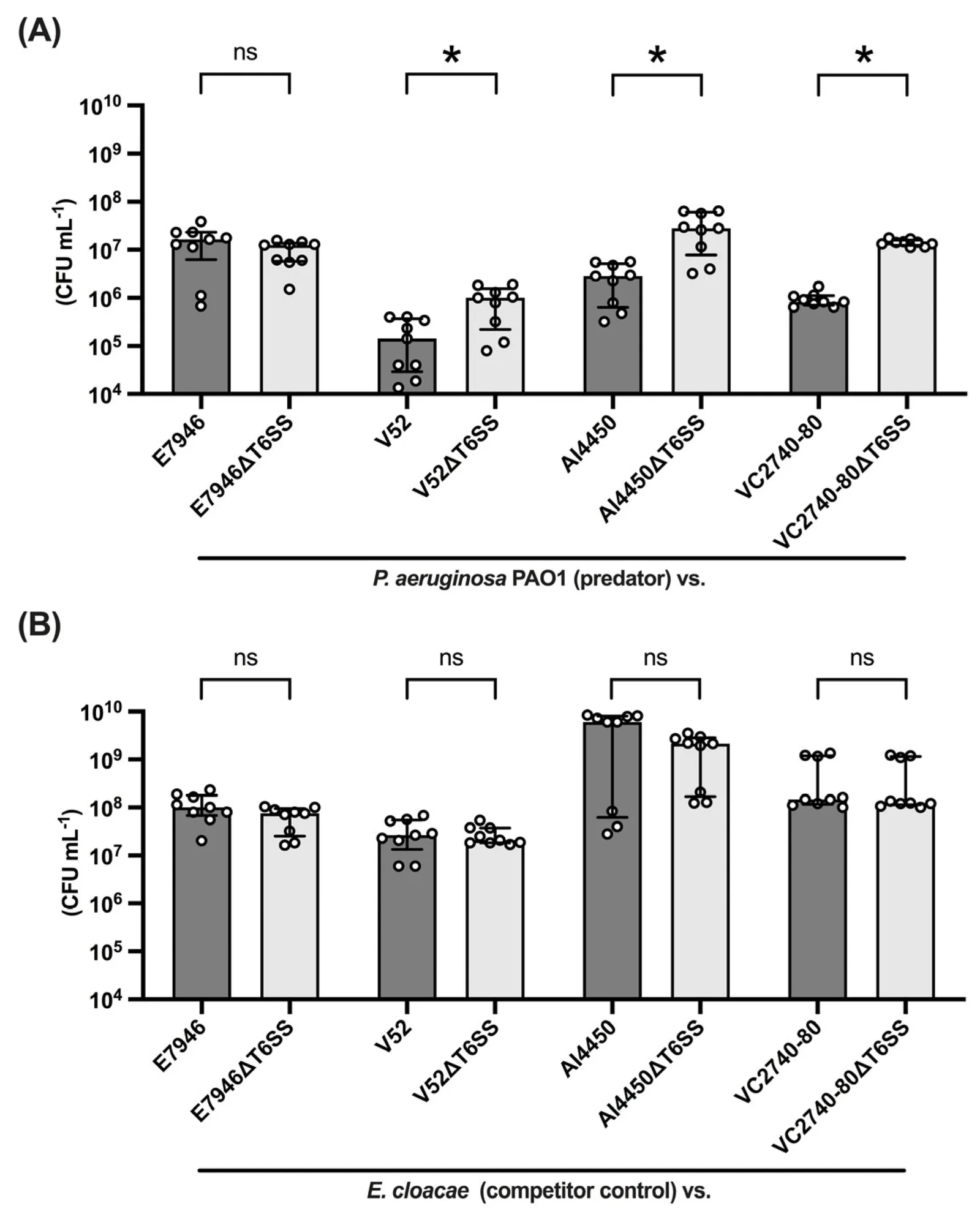
Survival assays of planktonic-derived *V. cholerae* strains with a T6SS-reactive *P. aeruginosa* as predator and the T6SS-unreactive *E. cloacae* as competitor control. Survival of planktonic-culture-derived *V. cholerae* wild-type isolates (light grey) as well as their isogenic ∆T6SS deletion mutants (dark grey) after co-incubation with (**A**) T6SS-reactive *P. aeruginosa* acting as predator or (**B**) *E. cloacae* serving as competitor control. Shown are the medians ± IQR of nine independent measurements. An asterisk indicates a significant difference in recovered CFUs between the T6SS^+^ wild-type *V. cholerae* isolate and its isogenic ∆T6SS mutant (*, *p* ≤ 0.05 using a Mann–Whitney U test; ns, *p* > 0.1 using a Mann–Whitney U test).

To assess the impact of T6SS activity on *V. cholerae* survival during inter-species competition, planktonic killing assays were conducted with *P. aeruginosa* as a T6SS-reactive competitor. Previous studies have shown that *P. aeruginosa* senses T6SS-mediated attacks from neighboring bacterial cells and responds by activating its own T6SS, engaging in retaliatory “tit-for-tat” behavior [36,37]. In line with previous reports describing T6SS-dependent killing by *P. aeruginosa*, the T6SS^+^ *V. cholerae* isolates V52, AI4450, and VC2740-80 exhibited significantly lower survival upon co-incubation with *P. aeruginosa* than their respective ∆T6SS deletion mutants (Figure 4). In contrast, no significant difference in survival was observed between T6SS^+^ E7946 and the corresponding ∆T6SS deletion mutant upon co-incubation with *P. aeruginosa* (Figure 4A). The distinct survival phenotype observed for E7946 points to considerable heterogeneity in susceptibility to T6SS-mediated competition among *V. cholerae* isolates. Such variation is consistent with previous studies demonstrating extensive diversity in T6SS regulation, effector repertoires, and immunity determinants across *V. cholerae* isolates [1,5,58].

To assess whether killing of T6SS^+^ *V. cholerae* was specifically driven by the potent T6SS of *P. aeruginosa*, co-incubation assays were repeated using *E. cloacae*. *E. cloacae* was included as a competitor control because its T6SS expression is reduced under LB growth conditions, suggesting limited T6SS-mediated predatory activity [39]. Thus, *E. cloacae* can be seen as a T6SS-independent competitor for nutrients in the assays. In contrast to the results obtained with the T6SS-reactive *P. aeruginosa*, no significant differences in survival upon co-incubation with *E. cloacae* were observed between T6SS^+^ *V. cholerae* isolates and their corresponding ∆T6SS deletion mutants. Thus, the lower survival rate upon exposure to *P. aeruginosa* is likely associated with T6SS-dependent counterattacks rather than general competition for nutrients or ecological niches.

Overall, these findings indicate that T6SS activity of *V. cholerae* can provoke a T6SS-dependent retaliatory response in *P. aeruginosa*, resulting in significant killing in three out of four T6SS^+^ *V. cholerae* isolates tested.

### 3.4. Silencing of the T6SS During Biofilm Formation Protects V. cholerae from P. aeruginosa-Mediated Killing

Due to the regulation of the T6SS core genes in *V. cholerae* indicating a significant reduction during biofilm formation, we hypothesized that biofilm-associated cells may exhibit an altered susceptibility to T6SS-dependent killing by *P. aeruginosa*. Thus, we continued with T6SS^+^ *V. cholerae* isolates V52, AI4450, and VC2740-80, which showed a significant susceptibility to the T6SS-dependent retaliatory response of *P. aeruginosa* during planktonic conditions. Killing assays with *P. aeruginosa* were repeated using the selected *V. cholerae* isolates grown under biofilm conditions.

**Figure 5:**
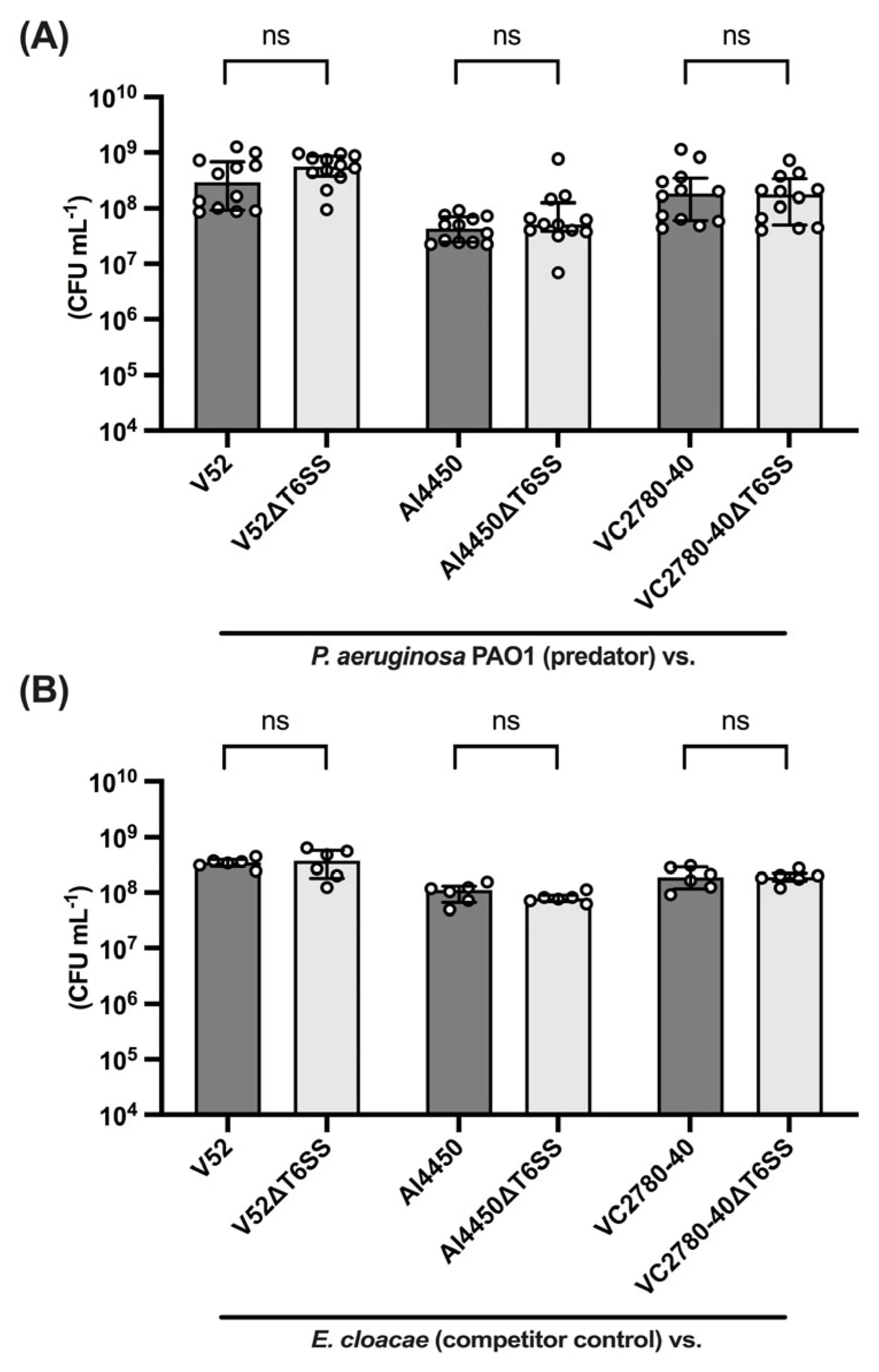
Survival assays of biofilm-derived *V. cholerae* strains with a T6SS-reactive *P. aeruginosa* as predator and the T6SS-unreactive *E. cloacae* as competitor control. Survival of biofilm-derived *V. cholerae* wild-type isolates (dark grey) as well as their isogenic ∆T6SS deletion mutants (light grey) after co-incubation with (**A**) T6SS-reactive *P. aeruginosa* acting as predator, or (**B**) *E. cloacae* serving as competitor control. Shown are (**A**) the medians ± IQR of 12 independent measurements with *P. aeruginosa* and (**B**) the medians ± IQR of six independent measurements with *E. cloacae*. Statistical analyses revealed no significant difference in recovered CFUs between the T6SS^+^ wild-type *V. cholerae* isolate and its isogenic ∆T6SS deletion mutant (ns, *p* > 0.1 using a Mann–Whitney U test).

When *V. cholerae* biofilms were co-incubated with *P. aeruginosa*, no significant differences in survival were detected between any T6SS^+^ *V. cholerae* isolate and its corresponding ∆T6SS deletion mutant (Figure 5A). Co-incubation of biofilm-derived *V. cholerae* with *E. cloacae* serving as a nutrient competitor revealed no significant differences in survival between T6SS^+^ strains and corresponding ∆T6SS mutants in any of the *V. cholerae* isolates tested (Figure 5B).

To exclude that the absence of T6SS-dependent killing during biofilm growth cannot be attributed to the physical barrier provided by the biofilm matrix, we additionally performed killing assays with biofilm-dispersed cells. To do so, the biofilm was mechanically disrupted using vortexing and treatment in an ultrasonic water bath, which results in detached cell aggregates of approximately 50–100 cells without affecting cell variability [52,57]. We selected *V. cholerae* VC2740-80 for these experiments because this isolate shows robust biofilm formation, significant downregulation of the main T6SS cluster and auxiliary cluster 1 during biofilm growth, and was previously characterized in the context of T6SS-mediated retaliatory response [37]. Killing assays with biofilm-dispersed cells revealed no significant difference in survival between the T6SS^+^ wild-type and the corresponding ∆T6SS deletion mutant (Figure S2). These results indicate that the biofilm matrix acting as a physical barrier for direct contact is not the primary cause for the observed protection against T6SS-dependent killing of *V. cholerae*-derived cells.

Taken together, these results indicate that the T6SS-dependent killing of planktonic-derived T6SS^+^ *V. cholerae* isolates by *P. aeruginosa* is abolished during biofilm growth [60,61]. This loss of susceptibility correlates with the expressional reduction in T6SS genes in *V. cholerae* during biofilm formation, indicating a strong link between reduced T6SS expression and enhanced resistance to T6SS-mediated counterattacks. These observations suggest that *V. cholerae* can conditionally downregulate T6SS activity during biofilm growth, potentially as an adaptive strategy to minimize the risk of retaliatory T6SS-dependent responses from competing bacteria, e.g., *P. aeruginosa*.

## 4. Discussion

Previous work demonstrated that two genes from the main T6SS cluster of *V. cholerae* exhibit reduced expression levels under biofilm-forming conditions, suggesting that T6SS activity is modulated during this stage of the pathogen’s life cycle [38]. Here, we demonstrate that the expression of the T6SS main cluster and auxiliary cluster 1 is generally repressed during biofilm formation relative to planktonic growth across diverse *V. cholerae* isolates. Notably, the expression levels differ considerably between isolates, indicating substantial variation in the baseline requirements for T6SS expression. The biofilm-associated repression of the T6SS main cluster and auxiliary cluster 1 appears to represent a conserved regulatory feature in *V. cholerae*. In contrast, regulation of auxiliary cluster 2 during biofilm growth varies among isolates, suggesting strain-specific regulatory mechanisms. Such variation likely reflects differences in genetic background and the adaptation of T6SS-mediated functions to distinct ecological niches.

In agreement with the biofilm-associated downregulation of several T6SS genes, deletion of the T6SS main cluster did not significantly affect biofilm formation in any of the diverse *V. cholerae* isolates tested. Nevertheless, biofilm biomass differed markedly between isolates, independent of their T6SS status. This pronounced variation suggests that biofilm formation in *V. cholerae* is primarily governed by isolate-specific genetic determinants and regulatory networks unrelated to T6SS activity.

The T6SS of *V. cholerae* is generally considered a preemptive antibacterial weapon, whereas *P. aeruginosa* predominantly employs a retaliatory “tit-for-tat” strategy, activating its T6SS upon attack and consequently killing T6SS^+^ *V. cholerae* [36,37]. Given that *V. cholerae* represses the T6SS during biofilm formation, we comparatively characterized the killing efficacy of a T6SS-reactive *P. aeruginosa* with *V. cholerae* derived from planktonic or biofilm growth conditions. Consistent with earlier reports, killing assays with planktonic *V. cholerae* revealed that an active T6SS provokes counterattacks by *P. aeruginosa*, which is detrimental to the majority of *V. cholerae* isolates. The susceptibility of *V. cholerae* observed in these assays was clearly T6SS-dependent, as ΔT6SS deletion mutants exhibited significantly increased survival following contact with *P. aeruginosa*. Furthermore, competition with the T6SS-unreactive strain *E. cloacae* [39] did not affect *V. cholerae* survival, irrespective of the cultivation, i.e., planktonic or biofilm-derived, or T6SS status, i.e., wild-type or ΔT6SS. Together, these findings indicate that the observed phenotype is driven by T6SS-mediated interbacterial interactions rather than by nonspecific effects of resource competition [39].

In contrast to planktonic cultures, biofilm-derived *V. cholerae* remain unaffected by *P. aeruginosa*. Thus, a key finding of this study reveals that biofilm-associated repression of T6SS genes correlates with the loss of T6SS-dependent killing of *P. aeruginosa*. Importantly, disruption of the biofilm matrix prior to exposure to *P. aeruginosa* did not restore a T6SS-dependent survival difference. While these results argue against the biofilm matrix being the primary explanation for the absence of T6SS-dependent killing under our experimental conditions, we cannot ultimately exclude that physiological changes in biofilm-associated cells may contribute to some level to the overall observed resistance phenotype. Based on the results provided herein, we suggest a model in which the repression of T6SS expression during biofilm growth enables *V. cholerae* to avoid eliciting retaliatory attacks from neighboring bacteria. Notably, T6SS deletion neither impaired biofilm formation nor affected survival under the tested conditions, suggesting that the observed phenotype arises directly from changes in T6SS activity rather than from biofilm-related properties themselves. The ecological relevance of the investigated inter-bacterial interactions is underscored by the fact that both *P. aeruginosa* and *E. cloacae* coexist with *V. cholerae* in aquatic environments and form multispecies biofilms [62,63,64].

Our findings indicate a conditional regulation of the T6SS in *V. cholerae*. While biofilm-dependent repression of T6SS expression in the main cluster and auxiliary cluster 1 is conserved in *V. cholerae*, the auxiliary cluster 2 seems to exhibit a more variable isolate-specific regulation. Our data demonstrate that an active T6SS can impose a substantial fitness cost for *V. cholerae* by provoking counterattacks from T6SS-reactive bacteria. Downregulation of the T6SS during biofilm growth may therefore constitute an adaptive strategy that enhances fitness in multispecies communities by reducing antagonistic interactions. Collectively, these findings broaden our understanding of the ecological role of the T6SS and highlight the importance of its regulation in balancing the advantages of interbacterial competition against the costs of provoking counterattacks across distinct ecological niches and stages of the *V. cholerae* life cycle.

## Figures and Tables

**Table 1:** Strains and plasmids used in this study.

Bacterial Strain	Description	Reference
***E. coli***		
DH5α*λpir*	*E.coli*, F^−^ *endA1 glnV44 thi-1 recA1 relA1 gyrA96 deoR nupG* Φ80∆*lacZ*ΔM15 Δ(*lacZYA*-*argF*) U169 *hsdR17*(_rK_^−^_mK_^+^) *λpir*RK6	[40]
SM10*λpir*	*E. coli, thi thr leu tonA lacY supE recA*::RPA-2-Te::Mu *λpir*, KmR	[41]
***V. cholerae***		
E7946	*V. cholerae* spontaneous streptomycin-resistant (SmR) mutant of E7946 (O1 El Tor Inaba, clinical isolate from Bahrain), *hapR*^+^ SmR	[42]
E7946ΔT6SS	Deletion of the main gene cluster of T6SS in E7946	This study
V52	*V. cholerae* spontaneous streptomycin-resistant (SmR) mutant of V52 (O37 clinical isolate from Sudan), SmR	[43]
V52ΔT6SS	Deletion of the main gene cluster of T6SS in V52	This study
AI4450	*V. cholerae* spontaneous streptomycin-resistant (SmR) mutant of AI4450 (O139 clinical isolate from Bangladesh), SmR	[44]
AI4450ΔT6SS	Deletion of the main gene cluster of T6SS in AI4450	This study
VC2740-80	*V. cholerae* spontaneous streptomycin-resistant (SmR) mutant of VC2740-80 (O1 El Tor clinical isolate from USA), SmR	[45]
VC2740-80ΔT6SS	Deletion of the main gene cluster of T6SS in VC2740-80	This study
E7946 VC_A0106::*phoA*	Insertion of pGP*phoA* downstream of VC_A0106 in E7946, SmR, ApR	This study
E7946 VC_A0124::*phoA*	Insertion of pGP*phoA* downstream of VC_A0124 in E7946, SmR, ApR	This study
E7946 VC_A0017::*phoA*	Insertion of pGP*phoA* downstream of VC_A0017 in E7946, SmR, ApR	This study
E7946 VC_1418::*phoA*	Insertion of pGP*phoA* downstream of VC_1418 in E7946, SmR, ApR	This study
V52 VC_A0106::*phoA*	Insertion of pGP*phoA* downstream of VC_A0106 in V52, SmR, ApR	This study
V52 VC_A0124::*phoA*	Insertion of pGP*phoA* downstream of VC_A0124 in V52, SmR, ApR	This study
V52 VC_A0017::*phoA*	Insertion of pGP*phoA* downstream of VC_A0017 in V52, SmR, ApR	This study
V52 VC_1418::*phoA*	Insertion of pGP*phoA* downstream of VC_1418 in V52, SmR, ApR	This study
AI4450 VC_A0106::*phoA*	Insertion of pGP*phoA* downstream of VC_A0106 in AI4450, SmR, ApR	This study
AI4450 VC_A0124::*phoA*	Insertion of pGP*phoA* downstream of VC_A0124 in AI4450, SmR, ApR	This study
AI4450 VC_A0017::*phoA*	Insertion of pGP*phoA* downstream of VC_A0017 in AI4450, SmR, ApR	This study
AI4450 VC_1418::*phoA*	Insertion of pGP*phoA* downstream of VC_1418 in AI4450, SmR, ApR	This study
VC2740-80 VC_A0106::*phoA*	Insertion of pGP*phoA* downstream of VC_A0106 in VC2740-80, SmR, ApR	This study
VC2740-80 VC_A0124::*phoA*	Insertion of pGP*phoA* downstream of VC_A0124 in VC2740-80, SmR, ApR	This study
VC2740-80 VC_A0017::*phoA*	Insertion of pGP*phoA* downstream of VC_A0017 in VC2740-80, SmR, ApR	This study
VC2740-80 VC_1418::*phoA*	Insertion of pGP*phoA* downstream of VC_1418 in VC2740-80, SmR, ApR	This study
E7946 *ompU*::*phoA*	Insertion of pGP*phoA* downstream of VC_0633 in E7946, SmR, ApR	[46]
V52 *ompU*::*phoA*	Insertion of pGP*phoA* downstream of VC_0633 in V52, SmR, ApR	This study
AI4450 *ompU*::*phoA*	Insertion of pGP*phoA* downstream of VC_0633 in AI4450, SmR, ApR	This study
VC2740-80 *ompU*::*phoA*	Insertion of pGP*phoA* downstream of VC_0633 in VC2740-80, SmR, ApR	This study
***P. aeruginosa*** **PAO1**	Prototrophic	[47]
***E. cloacae***	clinical isolate, collection Medical University of Graz	[48]
**Plasmids**		
pCVD442	*ori6K, mobRP4, sacB,* ApR	[49]
pΔT6SS	pCVD442 with upstream fragment of VC_A0105 and downstream fragment of VC_A0124 amplified from E7946, ApR	This study
pGP*phoA*	pGP704 with promotorless *phoA* of SM10*λpir*, ApR	[50]
pGP*phoA*-VC_A0106	pGP*phoA* with VC_A0106 fragment of E7946, ApR	This study
pGP*phoA*-VC_A0124	pGP*phoA* with VC_A0124 fragment of E7946, ApR	This study
pGP*phoA*-VC_A0017	pGP*phoA* with VC_A0017 fragment of E7946, ApR	This study
pGP*phoA*-VC_1418	pGP*phoA* with VC_1418 fragment of E7946, ApR	This study
pGP*phoA*-*ompU*	pGP*phoA* with VC_0633 fragment of E7946, ApR	[46]

**Table 2:** Oligonucleotides used in this study.

Primer	Sequence
T6SS_SacI_1	AAAGAGCTCAGTGAAACGAGTGAAGCAATC
T6SS_2	TAGTTCTCCCATTCTGTCCTCATCGGTTAGT
T6SS_3	AGGACAGAATGGGAGAACTAATTTGATTTTCAA
T6SS_SalI_4	AATGTCGACGATGAGAAATCCAAAGGTGAC
T6SS_col_1	AGAAACTGTTCAAGACACGTTA
T6SS_col_2	CATTGGATGACTGCAAAACATG
VC_A0106_SacI	ATTGAGCTCCCTGCGATAAGAAACTATATTC
VC_A0106_KpnI	AAAGGTACCATTATCCCAGTAAGCATCATGG
VC_A0124_SacI	TTAGAGCTCAATAACTTGCTTTCTGCGTATG
VC_A0124_KpnI	AAAGGTACCCTAACTATTATCAACATCCTCTA
VC_A0017_SacI	AAAGAGCTCTGAAGGCCATGAAGATGAGAT
VC_A0017_KpnI	AAAGGTACCCGCTTCGATTGGCTTACG
VC_1418_SacI	AAAGAGCTCCTGTTGCTATGGTAGATGGTT
VC_1418_KpnI	TTTGGTACCTCTTATTTGCACCTTGATTTCAT

Restriction sites are underlined.

## Data Availability

The original contributions presented in this study are included in the article/Supplementary Material. Further inquiries can be directed to the corresponding author.

## Supplementary Materials

The following supporting information can be downloaded at: https://www.mdpi.com/article/10.3390/microorganisms14092024/s1, Figure S1: Expression level of the outer membrane protein U (*ompU*). Reporter strains of *V. cholerae* E7946, V52, AI4450, and VC2740-80 were constructed using pGP*phoA-ompU* resulting in chromosomal fusion of the promotor region of VC_0633 to a promotorless *phoA* cassette. The PhoA activity indicates the individual expression level of *ompU* measured in “Miller units” from planktonic cultures (dark grey) and from biofilm cultures (light grey). Shown are the medians ± IQR of four independent measurements. An asterisk indicates differences relative to the planktonic cells compared to the biofilm cells (*, *p* < 0.05 using a Mann-Whitney U test; ns, *p* > 0.1 using a Mann-Whitney U test); Figure S2: Survival assays of biofilm-dispersed *V. cholerae* VC2740-80 with a T6SS-reactive *P. aeruginosa* as predator. Survival of biofilm-dispersed *V. cholerae* VC2740-80 (dark grey) as well as the isogenic ΔT6SS deletion mutant (light grey) after co-incubation with T6SS-reactive *P. aeruginosa* acting as predator. Shown are the medians ± IQR of eight independent measurements. No significant difference in recovered CFUs between the T6SS^+^ wildtype *V. cholerae* VC2740-80 compared to the isogenic ΔT6SS mutant was observed (ns, *p* > 0.2 using a Mann-Whitney U test).

## Abbreviations

The following abbreviations are used in this manuscript:

T6SS	Type VI secretion system
T6SS^+^	Strain expressing a functional type VI secretion system

## References

[1] Joshi A., Kostiuk B., Rogers A., Teschler J., Pukatzki S., Yildiz F.H. (2017). Rules of Engagement: The Type VI Secretion System in *Vibrio cholerae*. Trends Microbiol..

[2] Montero D.A., Vidal R.M., Velasco J., George S., Lucero Y., Gómez L.A., Carreño L.J., García-Betancourt R., O’Ryan M. (2023). *Vibrio cholerae*, classification, pathogenesis, immune response, and trends in vaccine development. Front. Med..

[3] Boyer F., Fichant G., Berthod J., Vandenbrouck Y., Attree I. (2009). Dissecting the bacterial type VI secretion system by a genome wide in silico analysis: What can be learned from available microbial genomic resources?. BMC Genom..

[4] Singh R.P., Kumari K. (2023). Bacterial type VI secretion system (T6SS): An evolved molecular weapon with diverse functionality. Biotechnol. Lett..

[5] Basler M., Pilhofer M., Henderson G.P., Jensen G.J., Mekalanos J.J. (2012). Type VI secretion requires a dynamic contractile phage tail-like structure. Nature.

[6] Pukatzki S., Ma A.T., Revel A.T., Sturtevant D., Mekalanos J.J. (2007). Type VI secretion system translocates a phage tail spike-like protein into target cells where it cross-links actin. Proc. Natl. Acad. Sci. USA.

[7] Felisberto-Rodrigues C., Durand E., Aschtgen M.-S., Blangy S., Ortiz-Lombardia M., Douzi B., Cambillau C., Cascales E. (2011). Towards a structural comprehension of bacterial type VI secretion systems: Characterization of the TssJ-TssM complex of an *Escherichia coli* pathovar. PLoS Pathog..

[8] Zoued A., Durand E., Santin Y.G., Journet L., Roussel A., Cambillau C., Cascales E. (2017). TssA: The cap protein of the Type VI secretion system tail. BioEssays.

[9] Planamente S., Salih O., Manoli E., Albesa-Jové D., Freemont P.S., Filloux A. (2016). TssA forms a gp6-like ring attached to the type VI secretion sheath. EMBO J..

[10] Brunet Y.R., Zoued A., Boyer F., Douzi B., Cascales E. (2015). The Type VI Secretion TssEFGK-VgrG Phage-Like Baseplate Is Recruited to the TssJLM Membrane Complex via Multiple Contacts and Serves As Assembly Platform for Tail Tube/Sheath Polymerization. PLoS Genet..

[11] Kube S., Kapitein N., Zimniak T., Herzog F., Mogk A., Wendler P. (2014). Structure of the VipA/B type VI secretion complex suggests a contraction-state-specific recycling mechanism. Cell Rep..

[12] Bröms J.E., Ishikawa T., Wai S.N., Sjöstedt A. (2013). A functional VipA-VipB interaction is required for the type VI secretion system activity of *Vibrio cholerae* O1 strain A1552. BMC Microbiol..

[13] Wood T.E., Howard S.A., Wettstadt S., Filloux A. (2019). PAAR proteins act as the ‘sorting hat’ of the type VI secretion system. Microbiology.

[14] Cianfanelli F.R., Alcoforado Diniz J., Guo M., Cesare V.d., Trost M., Coulthurst S.J. (2016). VgrG and PAAR Proteins Define Distinct Versions of a Functional Type VI Secretion System. PLoS Pathog..

[15] Shneider M.M., Buth S.A., Ho B.T., Basler M., Mekalanos J.J., Leiman P.G. (2013). PAAR-repeat proteins sharpen and diversify the type VI secretion system spike. Nature.

[16] Pukatzki S., Ma A.T., Sturtevant D., Krastins B., Sarracino D., Nelson W.C., Heidelberg J.F., Mekalanos J.J. (2006). Identification of a conserved bacterial protein secretion system in *Vibrio cholerae* using the Dictyostelium host model system. Proc. Natl. Acad. Sci. USA.

[17] Bönemann G., Pietrosiuk A., Diemand A., Zentgraf H., Mogk A. (2009). Remodelling of VipA/VipB tubules by ClpV-mediated threading is crucial for type VI protein secretion. EMBO J..

[18] Crisan C.V., Hammer B.K. (2020). The *Vibrio cholerae* type VI secretion system: Toxins, regulators and consequences. Environ. Microbiol..

[19] Zheng J., Ho B., Mekalanos J.J. (2011). Genetic analysis of anti-amoebae and anti-bacterial activities of the type VI secretion system in *Vibrio cholerae*. PLoS ONE.

[20] Altindis E., Dong T., Catalano C., Mekalanos J. (2015). Secretome analysis of *Vibrio cholerae* type VI secretion system reveals a new effector-immunity pair. mBio.

[21] Miyata S.T., Unterweger D., Rudko S.P., Pukatzki S. (2013). Dual expression profile of type VI secretion system immunity genes protects pandemic *Vibrio cholerae*. PLoS Pathog..

[22] West S.A., Griffin A.S., Gardner A., Diggle S.P. (2006). Social evolution theory for microorganisms. Nat. Rev. Microbiol..

[23] Matthey N., Stutzmann S., Stoudmann C., Guex N., Iseli C., Blokesch M. (2019). Neighbor predation linked to natural competence fosters the transfer of large genomic regions in *Vibrio cholerae*. eLife.

[24] Crisan C.V., Chande A.T., Williams K., Raghuram V., Rishishwar L., Steinbach G., Watve S.S., Yunker P., Jordan I.K., Hammer B.K. (2019). Analysis of *Vibrio cholerae* genomes identifies new type VI secretion system gene clusters. Genome Biol..

[25] Yanni D., Márquez-Zacarías P., Yunker P.J., Ratcliff W.C. (2019). Drivers of Spatial Structure in Social Microbial Communities. Curr. Biol..

[26] Zhao W., Caro F., Robins W., Mekalanos J.J. (2018). Antagonism toward the intestinal microbiota and its effect on *Vibrio cholerae* virulence. Science.

[27] McNally L., Bernardy E., Thomas J., Kalziqi A., Pentz J., Brown S.P., Hammer B.K., Yunker P.J., Ratcliff W.C. (2017). Killing by Type VI secretion drives genetic phase separation and correlates with increased cooperation. Nat. Commun..

[28] Borgeaud S., Metzger L.C., Scrignari T., Blokesch M. (2015). The type VI secretion system of *Vibrio cholerae* fosters horizontal gene transfer. Science.

[29] Miyata S.T., Kitaoka M., Brooks T.M., McAuley S.B., Pukatzki S. (2011). *Vibrio cholerae* requires the type VI secretion system virulence factor VasX to kill *Dictyostelium discoideum*. Infect. Immun..

[30] Fu Y., Waldor M.K., Mekalanos J.J. (2013). Tn-Seq analysis of *Vibrio cholerae* intestinal colonization reveals a role for T6SS-mediated antibacterial activity in the host. Cell Host Microbe.

[31] Fast D., Kostiuk B., Foley E., Pukatzki S. (2018). Commensal pathogen competition impacts host viability. Proc. Natl. Acad. Sci. USA.

[32] Logan S.L., Thomas J., Yan J., Baker R.P., Shields D.S., Xavier J.B., Hammer B.K., Parthasarathy R. (2018). The *Vibrio cholerae* type VI secretion system can modulate host intestinal mechanics to displace gut bacterial symbionts. Proc. Natl. Acad. Sci. USA.

[33] Dong T.G., Ho B.T., Yoder-Himes D.R., Mekalanos J.J. (2013). Identification of T6SS-dependent effector and immunity proteins by Tn-seq in *Vibrio cholerae*. Proc. Natl. Acad. Sci. USA.

[34] Zheng J., Shin O.S., Cameron D.E., Mekalanos J.J. (2010). Quorum sensing and a global regulator TsrA control expression of type VI secretion and virulence in *Vibrio cholerae*. Proc. Natl. Acad. Sci. USA.

[35] Ma A.T., Mekalanos J.J. (2010). *In vivo* actin cross-linking induced by *Vibrio cholerae* type VI secretion system is associated with intestinal inflammation. Proc. Natl. Acad. Sci. USA.

[36] Smith W.P.J., Brodmann M., Unterweger D., Davit Y., Comstock L.E., Basler M., Foster K.R. (2020). The evolution of tit-for-tat in bacteria via the type VI secretion system. Nat. Commun..

[37] Basler M., Ho B.T., Mekalanos J.J. (2013). Tit-for-tat: Type VI secretion system counterattack during bacterial cell-cell interactions. Cell.

[38] Pombo J.P., Ebenberger S.P., Müller A.M., Wolinski H., Schild S. (2022). Impact of Gene Repression on Biofilm Formation of *Vibrio cholerae*. Front. Microbiol..

[39] Soria-Bustos J., Ares M.A., Gómez-Aldapa C.A., González-Y-Merchand J.A., Girón J.A., La Cruz M.A.d. (2020). Two Type VI Secretion Systems of *Enterobacter cloacae* Are Required for Bacterial Competition, Cell Adherence, and Intestinal Colonization. Front. Microbiol..

[40] Platt R., Drescher C., Park S.K., Phillips G.J. (2000). Genetic system for reversible integration of DNA constructs and *lacZ* gene fusions into the *Escherichia coli* chromosome. Plasmid.

[41] Simon R., Priefer U., Pühler A. (1983). A Broad Host Range Mobilization System for In Vivo Genetic Engineering: Transposon Mutagenesis in Gram Negative Bacteria. Nat. Biotechnol..

[42] Miller V.L., DiRita V.J., Mekalanos J.J. (1989). Identification of *toxS*, a regulatory gene whose product enhances *toxR*-mediated activation of the cholera toxin promoter. J. Bacteriol..

[43] Boyd E.F., Waldor M.K. (2002). Evolutionary and functional analyses of variants of the toxin-coregulated pilus protein TcpA from toxigenic *Vibrio cholerae* non-O1/non-O139 serogroup isolates. Microbiology.

[44] Nair G.B., Ramamurthy T., Bhattacharya S.K., Mukhopadhyay A.K., Garg S., Bhattacharya M.K., Takeda T., Shimada T., Takeda Y., Deb B.C. (1994). Spread of *Vibrio cholerae* O139 Bengal in India. J. Infect. Dis..

[45] Goldberg S., Murphy J.R. (1983). Molecular epidemiological studies of United States Gulf Coast *Vibrio cholerae* strains: Integration site of mutator vibriophage VcA-3. Infect. Immun..

[46] Zingl F.G., Kohl P., Cakar F., Leitner D.R., Mitterer F., Bonnington K.E., Rechberger G.N., Kuehn M.J., Guan Z., Reidl J. (2020). Outer membrane vesiculation facilitates surface exchange and *in vivo* adaptation of *Vibrio cholerae*. Cell Host Microbe.

[47] Holloway B.W. (1955). Genetic recombination in *Pseudomonas aeruginosa*. J. Gen. Microbiol..

[48] Thapa H.B., Kohl P., Zingl F.G., Fleischhacker D., Wolinski H., Kufer T.A., Schild S. (2023). Characterization of the Inflammatory Response Evoked by Bacterial Membrane Vesicles in Intestinal Cells Reveals an RIPK2-Dependent Activation by Enterotoxigenic *Escherichia coli* Vesicles. Microbiol. Spectr..

[49] Donnenberg M.S., Kaper J.B. (1991). Construction of an eae deletion mutant of enteropathogenic *Escherichia coli* by using a positive-selection suicide vector. Infect. Immun..

[50] Moisi M., Jenul C., Butler S.M., New A., Tutz S., Reidl J., Klose K.E., Camilli A., Schild S. (2009). A novel regulatory protein involved in motility of *Vibrio cholerae*. J. Bacteriol..

[51] Horton R.M., Hunt H.D., Ho S.N., Pullen J.K., Pease L.R. (1989). Engineering hybrid genes without the use of restriction enzymes: Gene splicing by overlap extension. Gene.

[52] Seper A., Fengler V.H.I., Roier S., Wolinski H., Kohlwein S.D., Bishop A.L., Camilli A., Reidl J., Schild S. (2011). Extracellular nucleases and extracellular DNA play important roles in *Vibrio cholerae* biofilm formation. Mol. Microbiol..

[53] Manoil C. (1991). Analysis of membrane protein topology using alkaline phosphatase and beta-galactosidase gene fusions. Methods Cell Biol..

[54] Ebenberger S.P., Cakar F., Chen Y.C., Pressler K., Eberl L., Schild S. (2024). The activity of the quorum sensing regulator HapR is modulated by the bacterial extracellular vesicle (BEV)-associated protein ObfA of *Vibrio cholerae*. J. Extracell. Vesicles.

[55] MacIntyre D.L., Miyata S.T., Kitaoka M., Pukatzki S. (2010). The *Vibrio cholerae* type VI secretion system displays antimicrobial properties. Proc. Natl. Acad. Sci. USA.

[56] Mandakhalikar K.D., Rahmat J.N., Chiong E., Neoh K.G., Shen L., Tambyah P.A. (2018). Extraction and quantification of biofilm bacteria: Method optimized for urinary catheters. Sci. Rep..

[57] Tamayo R., Patimalla B., Camilli A. (2010). Growth in a biofilm induces a hyperinfectious phenotype in Vibrio cholerae. Infect. Immun..

[58] Unterweger D., Miyata S.T., Bachmann V., Brooks T.M., Mullins T., Kostiuk B., Provenzano D., Pukatzki S. (2014). The *Vibrio cholerae* type VI secretion system employs diverse effector modules for intraspecific competition. Nat. Commun..

[59] Crisan C.V., van Tyne D., Goldberg J.B. (2023). The type VI secretion system of the emerging pathogen *Stenotrophomonas maltophilia* complex has antibacterial properties. mSphere.

[60] Toska J., Ho B.T., Mekalanos J.J. (2018). Exopolysaccharide protects *Vibrio cholerae* from exogenous attacks by the type 6 secretion system. Proc. Natl. Acad. Sci. USA.

[61] Granato E.T., Smith W.P.J., Foster K.R. (2023). Collective protection against the type VI secretion system in bacteria. ISME J..

[62] Li X., Gu N., Huang T.Y., Zhong F., Peng G. (2022). *Pseudomonas aeruginosa*: A typical biofilm forming pathogen and an emerging but underestimated pathogen in food processing. Front. Microbiol..

[63] Misra T., Tare M., Jha P.N. (2022). Insights Into the Dynamics and Composition of Biofilm Formed by Environmental Isolate of *Enterobacter cloacae*. Front. Microbiol..

[64] Murga R., Miller J.M., Donlan R.M. (2001). Biofilm formation by gram-negative bacteria on central venous catheter connectors: Effect of conditioning films in a laboratory model. J. Clin. Microbiol..

