# Supplementary material for: Repression of the Type VI Secretion System (T6SS) During Biofilm Formation Provides a Fitness Advantage for Vibrio cholerae
Source: Microorganisms. 2026 Sep 11;14(9):2024. doi: 10.3390/microorganisms14092024 (PMC13609811; doi:10.3390/microorganisms14092024)
Supplement: Supplementary file 1 [file microorganisms-14-02024-s001.zip › microorganisms-4453793-supplementary.pdf]

## Supplementary materials

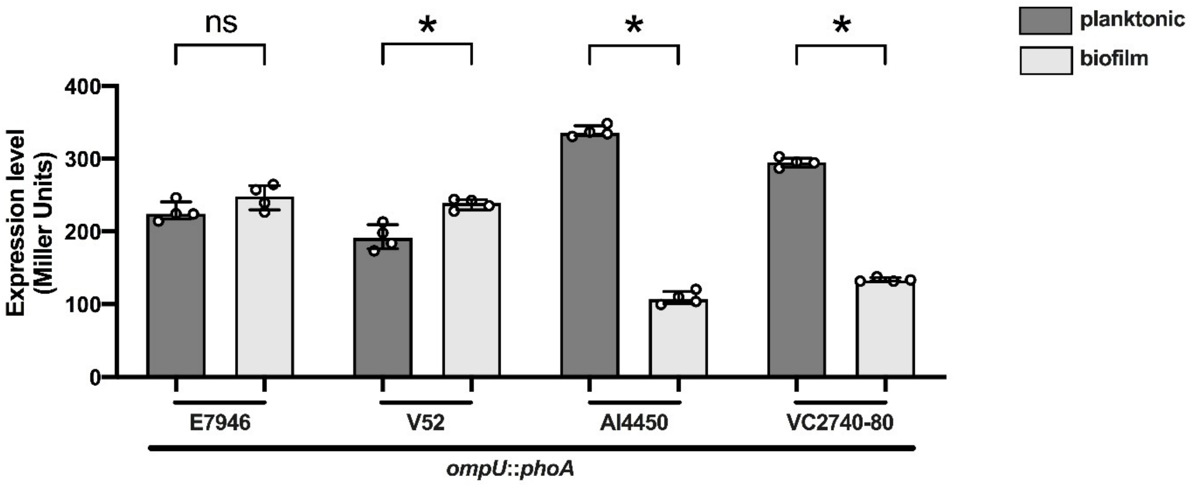


**Supplementary Figure S1.** Expression level of the outer membrane protein U (*ompU*). Reporter strains of *V. cholerae* E7946, V52, AI4450, and VC2740-80 were constructed using pGP*phoA-ompU* resulting in chromosomal fusion of the promotor region of VC_0633 to a promotorless *phoA* cassette. The PhoA activity indicates the individual expression level of *ompU* measured in "Miller units" from planktonic cultures (dark grey) and from biofilm cultures (light grey). Shown are the medians ± IQR of four independent measurements. An asterisk indicates differences relative to the planktonic cells compared to the biofilm cells (*, $p < 0.05$ using a Mann-Whitney U test; ns, $p > 0.1$ using a Mann-Whitney U test).

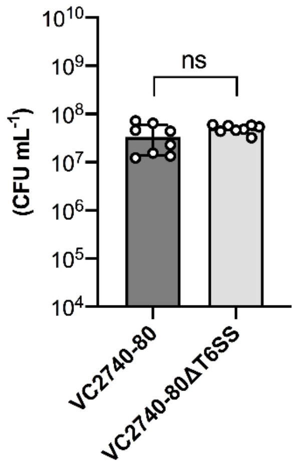


**Supplementary Figure S2.** Survival assays of biofilm-dispersed *V. cholerae* VC2740-80 with a T6SS-reactive *P. aeruginosa* as predator. Survival of biofilm-dispersed *V. cholerae* VC2740-80 (dark grey) as well as the isogenic ΔT6SS deletion mutant (light grey) after co-incubation with T6SS-reactive *P. aeruginosa* acting as predator. Shown are the medians ± IQR of eight independent measurements. No significant difference in recovered CFUs between the T6SS$^{+}$ wildtype *V. cholerae* VC2740-80 compared to the isogenic ΔT6SS mutant was observed (ns, $p > 0.2$ using a Mann-Whitney U test).
